# Patient-reported outcome 1 to 4 years after periprosthetic knee fracture: a nationwide cross-sectional matched study

**DOI:** 10.2340/17453674.2025.43083

**Published:** 2025-02-27

**Authors:** Stefan Kastalag RISAGER, Bjarke VIBERG, Charlotte Skov ABRAHAMSEN, Kristine Bollerup ARNDT, Anders ODGAARD, Martin LINDBERG-LARSEN

**Affiliations:** 1 Department of Orthopaedic Surgery and Traumatology, Odense University Hospital, Odense; 2 Department of Clinical Research, University of Southern Denmark, Odense; 3 Department of Orthopedic Surgery and Traumatology, Hospital Lillebaelt, University Hospital of Southern Denmark, Kolding; 4 Department of Regional Health Research, University of Southern Denmark, Odense; 5 Department of Orthopaedic Surgery and Traumatology, Rigshospitalet, Copenhagen; 6 Faculty of Health and Medical Sciences, Copenhagen University, Copenhagen, Denmark

## Abstract

**Background and purpose:**

Periprosthetic knee fractures (PPKFs) can be a serious complication after total knee arthroplasty (TKA). We aimed to compare patient-reported outcome (PRO) scores reported between 1 and 4 years after PPKF with a matched uncomplicated TKA control group.

**Methods:**

This nationwide cross-sectional matched cohort study included 372 TKA patients with a PPKF occurring from 2019 to 2022 and a control group of 878 uncomplicated TKA patients matched by age, time since TKA, and sex. The study population was derived from the Danish National Patient Register. The patients received questionnaires regarding knee function, quality of life, pain and satisfaction in 2023. The questionnaires included Oxford Knee Score (OKS), the Forgotten Joint Score (FJS), and the EQ-5D-5L Index.

**Results:**

The response rate was 48%. Mean OKS was 7 (confidence interval [CI] 5–9) points lower after a PPKF with a score of 30 (standard deviation [SD] 11) in the PPKF group vs 37 (SD 11) in the control group. The FJS was 13 (CI 7–19) points lower after a PPKF with a score of 50 (SD 30) in the PPKF group vs 63 (SD 30) in the control group. Mean EQ-5D-5L Index scores were 0.17 (CI 0.12–0.22) lower after a PPKF with a score of 0.68 (SD 0.25) in the PPKF group vs 0.85 (SD 0.25) in the control group. Additional analysis of patients who completed PROMs 1–2 years compared with 3–4 years after PPKF showed better PRO scores after 3–4 years with an OKS of 32 (SD 12) vs 27 (SD 12), FJS 55 (SD 32) vs 43 (SD 32), and EQ-5D-5L Index of 0.74 (SD 0.34) vs 0.60 (SD 0.34).

**Conclusion:**

Following PPKF, patients reported worse knee function, more pain, lower satisfaction, and poorer quality of life than those with uncomplicated TKAs.

Periprosthetic knee fracture (PPKF) after primary total knee arthroplasty (TKA) is a serious and potentially disabling complication. Treatment of a PPKF can be either operative or nonoperative and while a high overall reoperation rate has been reported [1,2], very little is known about the patient-reported outcome (PRO) [3-5]. Postoperative Oxford Knee Scores (OKS) 2–6 years after treatment with open reduction and internal fixation have been reported to range from 26 to 37 for femoral PPKFs and 32 to 40 for tibial PPKFs [3,5]. Additionally, patients with femoral PPKFs treated with revision TKA (rTKA) have shown OKS scores of 27 to 37 over the same follow-up period [3,4].

To assess outcomes after PPKF, a matched control group of uncomplicated TKA patients is needed, as it allows for measuring of the impact of PPKF alone. No studies have systematically compared PRO scores between PPKF patients and such a cohort, nor have they compared operatively and nonoperatively treated PPKF patients. Information on PROs after PPKF will enhance our understanding of PPKF and aid in managing patient expectations.

Therefore, our primary aim was to compare patient-reported outcomes between 1 and 4 years after PPKFs with a matched control group of uncomplicated primary TKAs in a cross-sectional study. Secondarily, we aimed to present patient-reported outcomes after a PPKF according to fracture site and operative vs nonoperative treatment.

## Methods

### Study design

This retrospective nationwide cross-sectional matched study was conducted and reported in accordance with STROBE [6]. It compares PROs following PPKF in patients with primary TKA with PROs of a matched control group of uncomplicated TKA patients through questionnaires. The questionnaires examined knee function, quality of life, pain, and satisfaction. As the questionnaires were distributed in 2023, PPKFs that occurred between 2019 and 2022 were included to assess patient-reported outcomes between 1 and 4 years after surgery. This timeframe captures patient status after the initial recovery from PPKF. The PPKF patients were identified and matched to the control group using a national registry.

### Participants

We included patients with a primary TKA based on extraction from the nationwide Danish National Patient Register (DNPR), from 1997 to 2022.

Exclusion criteria were unspecified TKA side, 2 primary TKAs in the same knee, emigration or death, or complication after TKA defined as ipsilateral infection, fracture prior to February 1, 2019, or reoperation (revision TKA [rTKA], arthrodesis, or amputation). Patients with bilateral TKAs were excluded from the control population, as we could not ensure which knee they would refer to when responding to the questionnaires.

Patient-reported outcome measures (PROMs) were distributed in 2 rounds. After the first round of distribution, the authors identified that including fracture-specific procedure codes significantly improved the sensitivity for identifying PPKFs, leading to a second round of distribution [7].

Patients with PPKF were matched by age, time with TKA, and sex to uncomplicated TKA patients defined as patients who did not have ipsilateral infection, fracture prior to February 1, 2019, or reoperation (rTKA, arthrodesis, or amputation) after their primary TKA. In the first round, patients were matched 3:1 to ensure sufficient power in the control group, but as the PPKF population grew, the ratio was adjusted to 2:1 in the second round to balance ethical considerations when requesting sensitive personal data. The first round of distribution was February 1, 2023 encompassing PPKFs 1–4 years prior (February 1, 2019, to February 1 2022) and PPKF was identified solely from diagnosis codes for fractures around the TKA in the distal femur, patella, or proximal tibia, classified using ICD-10 codes DS723, 724*, 820, 821*, and 822 [8]. The second round of distribution was October 1, 2023 encompassing PPKFs 1–4 years prior (October 1, 2019, to October 1, 2022) and PPKF was identified using both diagnosis codes and Nordic Medico-Statistical Committee (NOMESCO) procedure codes for fracture treatment around the TKA: procedure codes NFJ(%) (4–5) and NGJ(%) (0–2) [9]. The patients identified in the first round were not included in the second round.

### Data collection and management

All participants received access to electronic PROMs via a secure digital mailbox linked to their central person register (CPR) number. Reminders were sent for incomplete PROMs at 10, 20, and 60 days after the initial email. Partially completed questionnaires triggered a 60-day reminder. For those without a digital mailbox, questionnaires were sent by traditional mail. Data was included if at least 1 PROM was completed. Data collection was managed using the REDCap tool (https://project-redcap.org/) in collaboration with the Odense Patient Data Explorative Network (OPEN), Denmark [10].

### Data sources

The Danish Civil Registration System (CRS) allocates a unique identification number known as a CPR number to all persons who are either born or have been legal residents in Denmark for more than 3 months [11].

The Danish National Patient Register (DNPR) has recorded all in-hospital contacts in Denmark since 1977, and outpatient visits and emergency room data since 1995 [12]. The DNPR contains information on clinical characteristics such as procedure codes (NOMESCO) and diagnosis codes (ICD-10). The variables included age, time since TKA, sex, indication registered for primary TKA, osteoporosis (ICD-10 codes from 5 years prior), and Charlson Comorbidity Index (CCI) (ICD-10 codes from 10 years prior) [13].

### Outcomes

#### PROMS

Oxford Knee Score (OKS), the Forgotten Joint Score (FJS) and the Copenhagen Knee Range Of Motion Scale (CKRS) were used to assess knee function, while the EQ-5D-5L was used to evaluate generic health-related quality of life. In addition, supplementary questions regarding knee pain and satisfaction after a PPKF were also included in the assessment.

The OKS measures knee function after TKA and consists of 12 questions with a score of 0–48 points, 48 points being the best possible score. It has been translated into and validated in Danish [14]. Calculation of the OKS score followed the recommendations of the developers [15]. The minimal clinically important difference (MCID) for OKS is unknown after a PPKF, but reported MCIDs after TKA and rTKA were 5–8 [16,17].

The FJS is a 12-item PROM and measures to what degree an artificial knee joint is “forgotten.” It has a total score ranging from 0 to 100 with a high score indicating a high degree of “forgetting” the artificial knee joint. The FJS has been translated into Danish and the score was calculated using the recommendation of the developers [18]. The MCID for FJS is also unknown after a PPKF but has been reported to be 10–18 after TKA and rTKA [17,19].

The EQ-5D-5L Index and VAS measure health state and consist of a 5-item questionnaire and an EQ visual analogue score (VAS). EQ-5D-5L and VAS have been validated in Danish and the Danish utility scores were used when calculating EQ-5D-5L scores [20,21]. The MCID is unknown after a PPKF, and a uniform MCID is difficult to determine and is not appropriate due to variability in study methodologies, patient populations, and anchors [22].

The CKRS is a patient-reported estimate of the range of motion of a knee from a 2-item scale. Item 1 estimates the extension of the knee with 5 illustrations and a score of 0–5, 5 representing full extension and 0–3 an extension deficit with passive extension worse than 10°. Item 2 similarly estimates the flexion of the knee with 6 illustrations and a score of 0–6 with 6 being full flexion and 0–4 representing a flexion deficiency with passive flexion less than 100° [23]. No MCID exists for CKRS.

#### Pain

We asked the patients about their level of knee pain: “What was your average level of knee pain in the last month?” The answers were reported on a numeric rating scale (NRS) of 0–100 (0 = no pain, 100 = worst pain imaginable).

#### Satisfaction

We asked about satisfaction with the knee surgery: “How satisfied are you with the result after your knee surgery?” The answers were reported on an NRS scale of 0–100 (0 = very satisfied, 100 = not satisfied),” and “Are you, overall, satisfied with the result of your knee surgery? (Yes or No).”

### Statistics

No sample size was calculated. Missing data for each PROM was handled as recommended by the developers [15,18,21]. The data was analyzed according to guidelines for a structured manuscript [24].

Baseline characteristics for responders in both groups are presented as means with standard deviations (SD) or as numbers with percentages. The standard difference (std diff.) was calculated to compare the PPKF group with the control group.

The questionnaire responses were non-normally distributed and skewed due to floor or ceiling effects. However, given the large group sizes, the central limit theorem was applied [25]. Data was presented as crude means and adjusted means with SDs. Adjusted means were adjusted for osteoporosis and CCI and calculated using ordinary least squares (OLS) regression for continuous variables, a generalized linear model (GLM) for categorical variables, and logistic regression for binary variables. The differences between groups were determined based on the adjusted means and reported with a 95% confidence interval (CI). The subgroups were divided based on fracture site and treatment type (operatively treated, including rTKA within 30 days of fracture, vs nonoperatively treated patients). However, the subgroups were too small to allow for statistical comparison.

In an additional analysis, PPKF patients were divided into those who completed PROMs 1 to 2 years after their PPKF and those who completed them 3 to 4 years later. Kernel density curves were used to visualize the distribution of OKS, FJS, and pain scores between the PPKF and control groups, as well as between the 2 time-based PPKF subgroups. All analyses were performed using Stata Statistical Software version 18.0 (StataCorp LLC, College Station, TX, USA).

### Ethics, funding, use of AI, and disclosures

The study was approved by the data-responsible authority, the Region of Southern Denmark (reference no. 22/17275). Patient contact information was provided by the Danish Health Data Authority. Permission to access DNPR data after PROM completion was obtained from all participants. The study was funded by the Danish Rheumatism Association, Odense University Hospital, and Rigshospitalet. ChatGPT was used to improve the text’s linguistic, grammatical, and typographical aspects. The authors declare no conflicts of interest. Complete disclosure of interest forms according to ICMJE are available on the article page, doi: 10.2340/17453674.2025.43083

## Results

**Figure 1:**
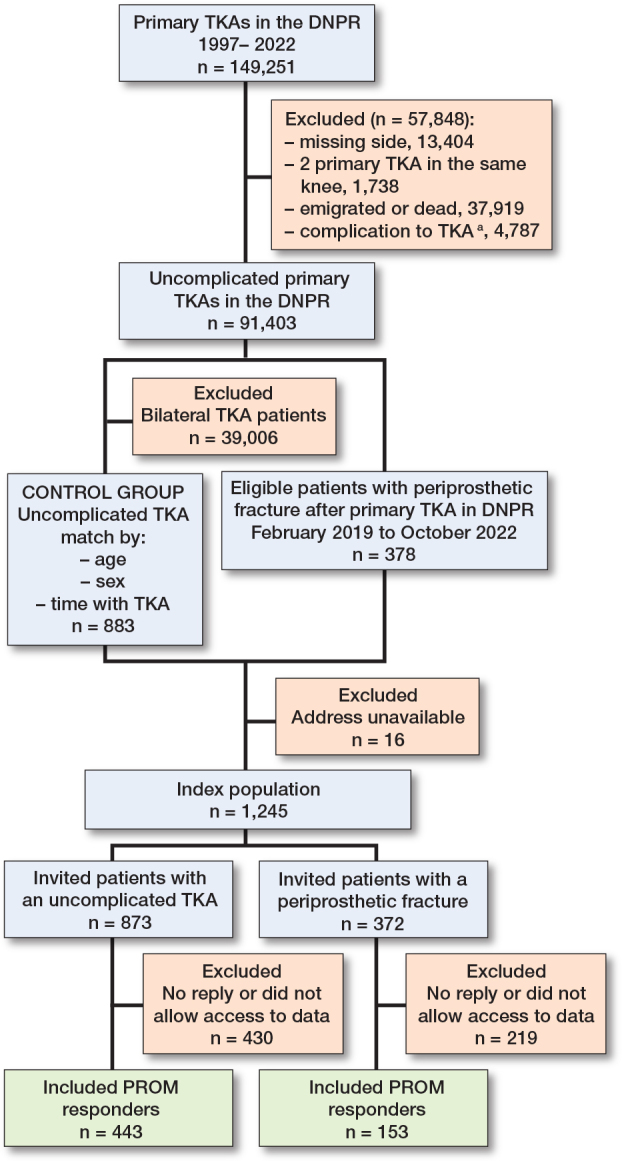
Flowchart of patients included/excluded in this study. DNPR = Danish National Patient Register; TKA = total knee arthroplasty; PROM = patient-reported outcome measure. **^a^** Complication include: ipsilateral infection, fracture prior to February 1, 2019, or reoperation (revision TKA, arthrodesis, or amputation).

The initial cohort included 149,251 primary TKAs performed between 1997 and 2022. After excluding 57,848 TKAs, 91,403 uncomplicated TKAs remained. Among these, 378 PPKF cases were identified between February 1, 2019, and October 1, 2022. An additional 39,006 bilateral TKAs were excluded before matching, and 883 controls were successfully matched (Figure 1). The response rate was 48% (41% in the PPKF group and 51% in the control group), with response from 596 (153 with PPKF and 443 controls) of the 1,245 included patients (Figure 1). The groups were similar across the matching criteria and indications for TKA; however, the PPKF group had a higher number of participants with osteoporosis (12% vs 5%) and higher CCI (1.1 (SD 1.2) vs 0.9 (SD 0.9) (Table 1).

**Table 1:** Demographic characteristics of 153 responders to PROMs with a periprosthetic knee fracture and 443 responders to PROMs with uncomplicated TKA. Values are count (%) unless otherwise specified

Characteristics	Responders		Std diff.
	PPKF	Uncomplicated TKA	
Included responders, n	153	443	
Mean age, years (SD)	65 (11)	65 (10)	< 0.001
Mean time with TKA, years (SD)	9 (6)	9 (6)	0.01
Women	110 (72)	311 (70)	0.04
Osteoporosis	19 (12)	20 (4.5)	0.29
Mean CCI (SD)	1.1 (1.2)	0.9 (0.9)	0.24
Indication for TKA			0.12
Osteoarthritis	128 (84)	391 (88)	
Previous fracture	5 (3.3)	12 (2.7)	
Rheumatoid arthritis	6 (3.9)	9 (2.0)	
Other/unknown	14 (9.2)	31 (7.0)	

PROM = patient reported outcome measure; PPKF = periprosthetic knee fracture; TKA = total knee arthroplasty; Std diff = standard difference; SD = standard deviation; CCI = Charlson Comorbidity Index.

### Outcomes

#### Knee function

**Figure 2:**
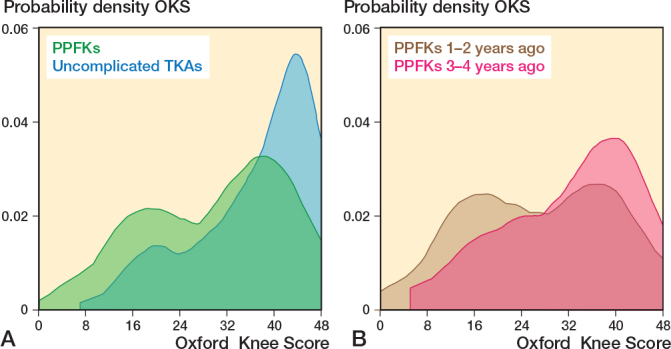
A. Kernel density score for Oxford Knee Score in 153 patients with a periprosthetic knee fracture (PPKF) and 439 patients with uncomplicated total knee arthroplasty (TKA). The y-axis shows data concentration, with the total curve area equal to 1, and areas between points representing data proportions. B. The PPKF group were further divided into 71 patients with PPKF 1–2 years ago and 82 patients 3–4 years ago.

**Figure 3:**
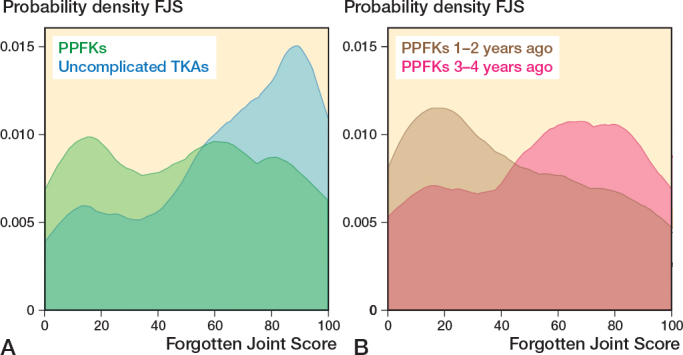
A. Kernel density score for Forgotten Joint Score in 134 patients with a PPKF and 432 patients with uncomplicated TKA. B. The PPKF group were further divided into 63 patients with PPKF 1–2 years ago and 71 patients 3–4 years ago. For abbreviations, see Figure 2.

Mean OKS score difference was 7 (CI 5–9) points, worse after PPKF with a score of 30 (SD 11) vs 37 (SD 11) in the control group. FJS score difference was 13 (CI 7–19) points, worse after PPKF with a score of 50 (SD 30) vs 63 (SD 30) in the control group (Table 2 and Figures 2 and 3). The PPKF group had a 0.13 (CI 0.04–0.21) higher probability of having a flexion deficit, with a probability of 0.32 (SD 0.46) compared with 0.19 (SD 0.39) for the control group. The probability for extension deficit was 0.13 (CI 0.05–0.21) higher for the PPKF group with a probability of 0.31 (SD 0.46) compared with 0.18 (SD 0.38) for the control group. The differences were statistically significant (Table 2).

**Table 2:** Patient-reported outcome of 153 patients with a PPKF vs a control group of 443 patients with uncomplicated TKA. Values are mean (SD) unless otherwise specified

Patient-reported outcome	PPKF			Uncomplicated TKA			Adjusted difference (CI)
	Crude	Adjusted	n ^a^	Crude	Adjusted	n ^a^	
Oxford Knee Score (0–48) **^b^**	30 (12)	30 (11)	153	37 (10)	37 (11)	439	7 (5 to 9)
Forgotten Joint Score (0–100) **^b^**	50 (33)	50 (30)	134	63 (31)	63 (30)	432	13 (7 to 19)
Pain (0–100, 0 = no pain)	34 (31)	34 (27)	145	21 (25)	21 (27)	433	–12 (–17 to –7)
EQ-5D-5L Index (0–1) **^b^**	0.67 (0.34)	0.68 (0.25)	144	0.85 (0.22)	0.85 (0.25)	429	0.17 (0.12 to 0.22)
EQ VAS (0–100) **^b^**	57 (28)	57 (27)	144	67 (26)	67 (26)	428	10 (5 to 15)
Satisfaction (0–100, 0 = satisfied)	34 (35)	33 (33)	145	22 (32)	23 (32)	431	–11 (–17 to –4)
Satisfied with treatment, n (%) **^c^**	86 (59)	0.59 (0.50)	146	346 (80)	0.80 (0.40)	433	0.21 (0.12 to 0.30)
CKRS							
Flexion (0–6)	4.7 (1.3)	4.7 (1.2)	147	5.1 (1.1)	5.1 (1.2)	434	0.4 (0.2 to 0.6)
Flexion deficit (0–4) , n (%) **^c^**	48 (31)	0.32 (0.46)	147	86 (19)	0.19 (0.39)	434	–0.13 (–0.21 to –0.04)
Extension (0–5)	3.7 (1.1)	3.7 (1.0)	147	4.1 (0.9)	4.0 (1.0)	434	0.3 (0.2 to 0.5)
Extension deficit (0–3), n (%) **^c^**	49 (32)	0.31 (0.46)	147	81 (18)	0.18 (0.38)	434	–0.13 (–0.21 to –0.05)

For Abbreviations, see Table 1, and CI = 95% confidence interval; CKRS = Copenhagen Knee Range Of Motion Scale.

a Responders to each questionnaire.

b Higher is better.

c Adjusted means calculated using logistic regression and presented as values between 0 and 1, representing probabilities.

Across fracture locations and treatments, operatively treated femoral and patellar PPKFs had the lowest OKS scores (27 [SD 12]) and operatively treated patellar PPKFs also had the lowest FJS scores at 31 (SD 31). In contrast, nonoperatively treated tibial PPKFs had the highest OKS score of 34 (SD 12) and FJS score of 63 (SD 30). Operatively treated femoral PPKFs showed the highest probability of flexion and extension deficits (0.37 [SD 0.49] and 0.40 [SD 0.49]), while nonoperatively treated femoral PPKFs had the lowest extension deficit (0.22 [SD 0.42]) and operatively treated patellar PPKFs reported no extension deficit (Tables 3–5).

**Table 3:** Patient-reported outcome of 67 operatively treated and 36 nonoperatively treated patients with femoral PPKF. Values are mean (SD) unless otherwise specified

Patient-reported outcome	Operatively treated			Nonoperatively treated		
	Crude	Adjusted	n ^a^	Crude	Adjusted	n ^a^
Oxford Knee Score	27 (12)	27 (12)	67	31 (12)	31 (13)	36
Forgotten Joint Score	42 (31)	44 (34)	55	57 (35)	55 (34)	33
Pain	39 (32)	39 (32)	63	31 (31)	32 (31)	34
EQ-5D-5L Index	0.62 (0.36)	0.61 (0.36)	63	0.69 (0.35)	0.71 (0.36)	34
EQ VAS	56 (26)	56 (29)	63	53 (31)	52 (29)	34
Satisfaction	40 (34)	41 (36)	63	32 (36)	32 (36)	34
Satisfied with						
treatment, n (%) **^c^**	34 (53)	0.54 (0.52)	64	19 (56)	0.55 (0.52)	34
CKRS						
Flexion	4.5 (1.3)	4.5 (1.4)	65	4.7 (1.5)	4.7 (1.4)	34
Flexion deficit, n (%) **^c^**	25 (37)	0.37 (0.49)	65	12 (33)	0.33 (0.48)	34
Extension	3.5 (1.2)	3.5 (1.1)	65	3.9 (1.0)	3.9 (1.2)	34
Extension deficit, n (%) **^c^**	27 (40)	0.40 (0.49)	65	8 (22)	0.22 (0.42)	34

See Table 2.

**Table 4:** Patient-reported outcome of 8 operatively treated and 26 nonoperatively treated patients with patellar PPKF. Values are mean (SD) unless otherwise specified

Patient-reported outcome	Operatively treated			Nonoperatively treated		
	Crude	Adjusted	n ^a^	Crude	Adjusted	n ^a^
Oxford Knee Score	27 (15)	27 (12)	8	33 (12)	33 (12)	26
Forgotten Joint Score	33 (28)	31 (31)	7	60 (29)	60 (30)	24
Pain	35 (26)	39 (31)	8	29 (30)	28 (29)	25
EQ-5D-5L Index	0.60 (0.39)	0.68 (0.29)	8	0.74 (0.29)	0.72 (0.28)	25
EQ VAS)	51 (21)	51 (30)	8	58 (30)	58 (29)	25
Satisfaction	28 (32)	27 (35)	8	26 (36)	28 (33)	25
Satisfied with						
treatment, n (%) **^c^**	5 (63)	0.67 (0.54)	8	20 (80)	0.80 (0.43)	25
CKRS						
Flexion	5.5 (0.5)	5.4 (0.9)	8	5.1 (1.0)	5.1 (0.9)	25
Flexion deficit, n (%) **^c^**	0 (0)	0	8	6 (23)	0.20 (0.45)	25
Extension	3.9 (1.4)	3.9 (1.2)	8	4.0 (1.0)	3.9 (1.1)	25
Extension deficit, n (%) **^c^**	3 (38)	0.31 (0.54)	8	6 (23)	0.23 (0.45)	25

See Table 2.

**Table 5:** Patient-reported outcome of 9 operatively treated and 7 nonoperatively treated patients with tibial PPKF. Values are mean (SD) unless otherwise specified

Patient-reported outcome	Operatively treated			Nonoperatively treated		
	Crude	Adjusted	n ^a^	Crude	Adjusted	n ^a^
Oxford Knee Score	31 (11)	31 (12)	9	34 (9)	34 (12)	7
Forgotten Joint Score	44 (30)	44 (30)	8	63 (40)	63 (30)	7
Pain	33 (35)	31 (29)	8	12 (25)	14 (30)	7
EQ-5D-5L Index	0.72 (0.27)	0.73 (0.23)	8	0.83 (0.17)	0.82 (0.24)	6
EQ VAS	72 (23)	73 (23)	8	70 (30)	69 (24)	6
Satisfaction	28 (32)	28 (27)	8	15 (25)	16 (27)	7
Satisfied with						
treatment, n (%) **^c^**	5 (63)	0.62 (0.52)	8	3 (43)	0.44 (53)	7
CKRS						
Flexion	4.9 (0.8)	4.8 (1.0)	8	5.1 (1.2)	5.2 (1.0)	7
Flexion deficit, n (%) **^c^**	3 (33)	0.41 (0.59)	8	2 (29)	0.17 (0.52)	7
Extension	3.5 (1.1)	3.4 (1.2)	8	3.9 (1.1)	3.9 (1.2)	7
Extension deficit, n (%) **^c^**	3 (33)	0.43 (0.55)	8	2 (29)	0.18 (0.44)	7

See Table 2.

#### Health status

Mean EQ-5D-5L Index score difference was 0.17 (CI 0.12–0.22), lower after PPKF with a score of 0.68 (SD 0.25) vs 0.85 (SD 0.25) for the control group. EQ VAS score difference was 10 (CI 5–15) points, lower after PPKF with a score of 57 (SD 27) vs 67 (SD 26) for the control group (Table 2). The differences were statistically significant (Table 2).

Across fracture location and treatment options (Tables 3–5), operatively treated femoral PPKFs had the lowest EQ-5D-5L index score of 0.61 (SD 0.36) and operatively treated patellar fractures had the lowest EQ VAS 51 (SD 30). Nonoperatively treated tibial PPKFs had the highest EQ-5D-5L Index and EQ VAS scores of 0.82 (SD 0.24) and 69 (SD 24) respectively.

#### Pain and satisfaction

**Figure 4:**
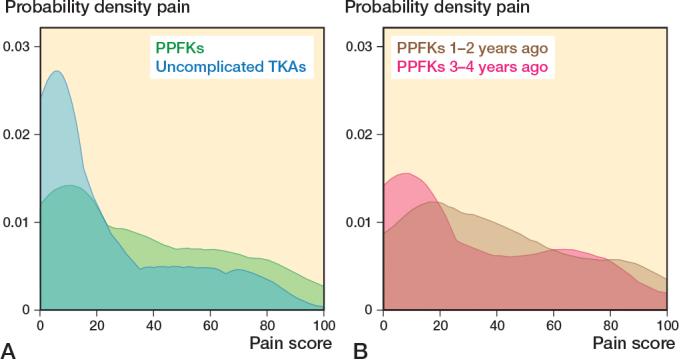
A. Kernel density score for pain in 145 patients with a PPKF and 433 patients with uncomplicated TKA. B. The PPKF group were further divided into 69 patients with PPKF 1–2 years ago and 76 patients 3–4 years ago. For abbreviations, see Figure 2.

Mean pain was 17 (CI 7–17) points worse after PPKF with a score of 34 (SD 27) in the PPKF group and 21 (SD 27) in the control group (Table 2 and Figure 4). Mean satisfaction was 11 (CI 4–17) points worse after a PPKF with a score of 33 (SD 33) in the PPKF group compared with 23 (SD 32) in the control group. The probability of overall satisfaction with the treatment was 0.21 (CI 0.12–-0.30) lower for the PPKF group with a probability of 0.59 (SD 0.50) compared twith 0.80 (SD 0.40) in the control group (Table 2).

Across fracture locations and treatments (Tables 3–5), the lowest reported pain was after nonoperatively treated tibial fractures (14 [SD 30]) and highest after operatively treated patellar and femoral PPKFs (39 [SD 31 and 32 respectively]). The highest probability of satisfaction was reported after nonoperatively treated patellar PPKFs (0.80 [SD 0.42]) and the lowest after nonoperatively treated tibial PPKFs (0.44 [SD 0.53]).

### Additional analysis

Patients who experienced a PPKF within 2 years before completing the PROMs had worse scores across all parameters compared with those experiencing a PPKF 2 to 4 years before completing the PROMs (Table 6 and Figures 2–4). To determine whether the difference was attributable to fracture sites and treatment, we tested these factors and found that they were evenly distributed between 2 time periods (Table 6).

**Table 6:** Patient-reported outcome of 153 patients with a PPKF divided according to time of fracture. Values are mean (SD) unless otherwise specified

Patient-reported outcome	PPKF 1–2 years ago			PPKF 3–4 years ago			Adjusted difference (CI)
	Crude	Adjusted	n ^a^	Crude	Adjusted	n ^a^	
Oxford Knee Score	27 (12)	27 (12)	71	32 (11)	32 (12)	82	5 (1 to 9)
Forgotten Joint Score	43 (32)	43 (32)	63	55(32)	55 (32)	71	12 (1 to 24)
Pain	39 (31)	40 (31)	69	29 (30)	28 (31)	76	–11 (–22 to 1)
EQ-5D-5L	0.61 (0.37)	0.60 (0.34)	68	0.73 (0.30)	0.74 (0.34)	76	0.13 (0.02 to 0.25)
EQ VAS	52 (28)	52 (28)	68	61 (27)	61 (28)	76	9 (0 to 19)
Satisfaction	38 (34)	39 (35)	69	30 (35)	29 (35)	76	–9 (–29 to 2)
Satisfied with							
treatment, n (%) **^c^**	34 (49)	0.49 (0.51)	69	52 (68)	0.68 (0.48)	77	0.19 (0.03 to 0.36)
CKRS							
Flexion	4	4.5 (1.3)	70	4	4.9 (1.3)	77	0.4 (0.0 to 0.8)
Flexion deficit, n (%) **^c^**	27 (38)	0.39 (0.49)	70	22 (27)	0.23 (0.41)	77	–0.15 (–0.30 to 0.00)
Extension	5	3.6 (1.1)	70	5	3.8 (1.1)	77	0.2 (–0.2 to 0.6)
Extension deficit, n (%) **^c^**	28 (39)	0.38 (0.49)	70	20 (24)	0.26 (0.43)	77	–0.12 (–0.27 to 0.03)

See Table 2.

For the patients with PPKF 1–2 years ago, 56% were operatively treated (48% femoral, 4% patella, and 4% tibia), and 44% were nonoperatively treated (25% femur, 16% patella, and 3% tibia).

For the patients with PPKF 3–4 years ago, 54% were operatively treated (40% femoral, 6% patella, and 8% tibia) and 46% were nonoperatively treated (22% femur, 18% patella, and 6% tibia).

## Discussion

We aimed to compare patient-reported outcome (PRO) scores reported between 1 and 4 years after PPKF with a matched uncomplicated TKA control group. We showed that the PPKF group reported significantly lower knee scores, experienced more pain, and had lower satisfaction.

### Knee function

The mean OKS of 30 after a PPKF is in line with previous studies [3-5]. As expected, the mean OKS and FJS scores were lower in the PPKF group than in the uncomplicated TKA group. The MCID for this patient group is unknown, so the clinical significance of the difference cannot be definitively determined. However, the reported MCIDs for TKA and rTKA were 5–8 for OKS and 10–18 for FJS [16,17,19]. In our study, the observed differences were 7 (CI 5–9) for OKS and 13 (CI 7–19) for FJS, suggesting potential clinical significance. More patients in the PPKF group also had both flexion and extension deficits. A similarly study designed by Arndt et al. [26] reported an OKS score of 25 and 31 after rTKA due to unexplained pain and aseptic loosening 1 to 3 years after revision surgery, respectively. These findings suggest that while a PPKF may be challenging to treat and could require additional surgery [2], the patient-reported outcome might be comparable to those of rTKA in general.

The OKS and FJS for the PPKF group show 2 distinct peaks. The peak representing lower PRO scores is especially noticeable for PROMs completed 1–2 years after PPKF, while the peak representing higher PRO scores is more noticeable for PROMs completed 3–4 years after PPKF. These results could suggest that recovery for some PPKF patients might be prolonged, with knee function continuing to improve over the first few years.

Across fracture locations and treatments, the sample sizes were too small to draw definitive conclusions. However, operatively treated PPKFs were associated with lower knee function scores than nonoperatively treated PPKFs, suggesting these injuries may be more severe and challenging to recover from. The severity of the fracture is unknown in this study, and the difference should not be attributed to the choice of treatment. Operatively treated patellar PPKF patients reported very poor knee function. Despite the small sample (8 patients), this aligns with the previously reported high reoperation rate (46%) [2].

### Health status, pain, and satisfaction

The mean EQ-5D-5L index, satisfaction, and pain in the PPKF group was lower than that observed in the control group. The reduced EQ-5D-5L indicates decrease in quality of life following a PPKF. Although the control group was matched, the PPKF group had a higher mean CCI; however, the difference remained when adjusting for CCI. The EQ-5D-5L scores vary among different populations, and the Danish population typically exhibits a high EQ-5D-5L mean score of 0.90 [20]. The EQ-5D-5L score in the control group is close to that of the Danish population, while the scores for the PPKF group were comparable to the score after rTKA due to aseptic loosening reported by Arndt et al. [26].

### Limitations

The nationwide setting provides a large sample reflecting the population suffering from PPKFs. However, the 48% response rate and missing data introduce uncertainty about how accurately the true PPKF population is represented. DNPR data for non-responders may not be accessed without their consent; as a result, their baseline characteristics remain unknown. The statistical inference is valid assuming missing data can be assumed to be “Missing Completely At Random” (MCAR) [24]. However, reasons for non-participation for 47 patients included dementia, Parkinson’s disease, immobilization, grief, electronic difficulties, and recent death. Therefore, the absence of these patients might bias the observed PRO scores.

Another limitation is the lack of MCID and baseline PRO scores prior to the PPKFs. Without MCID we cannot interpret whether the differences are clinically relevant, and without baseline data the delta change in the PPKF group is unknown. However, baseline PRO may be very difficult to obtain and of limited value as the functional status at the time of an acute fracture is very poor and difficult for the patients to report.

The PPKF patients were initially matched 3:1, then 2:1, possibly better representing the PPKF patients identified in the first round. The PPKF group had a higher percentage of patients with osteoporosis and a high CCI. Additionally, fracture classification, prosthesis stability, status of the extensor apparatus, patella resurfacing, and treatment choice were unknown. Therefore, caution is advised when interpreting the findings, as only overall estimates of patient-reported outcomes are provided.

Any difference outcome likely reflects fracture severity more than the treatment itself. The information in this study should therefore be used to manage patient expectations rather than to guide choice of treatment.

### Conclusion

Patients following PPKF reported worse knee function, more pain, lower satisfaction, and poorer quality of life compared with matched patients with uncomplicated TKA. The PPKF patients who completed questionnaires 3–4 years after their PPKF reported better knee function and greater satisfaction than those who completed them 1–2 years after, suggesting a lengthy recovery process for some patients. Operatively treated PPKF patients had worse PRO scores than those treated nonoperatively, particularly after patellar PPKFs.

## Notes

SKR, BV, CA, KBB, AO, and MLL conceptualized the study. SKR handled data curation, analysis, and project management, supervised by MLL and BV. SKR wrote the original draft, with BV, CA, KBB, AO, and MLL contributing to review and editing.

The authors would like to thank Odense Patient data Explorative Network (OPEN) for their contribution on data management and service in REDCap.

Handling co-editors: Marianne Westberg and Robin Christensen

*Acta* thanks Ove Nord Furnes and Annette W-Dahls for help with peer review of this manuscript.
